# The Dry Sliding Wear Properties of Nano-Sized TiC_p_/Al-Cu Composites at Elevated Temperatures

**DOI:** 10.3390/ma10080939

**Published:** 2017-08-11

**Authors:** Wei-Si Tian, Qing-Long Zhao, Chuan-Jiang Zhao, Feng Qiu, Qi-Chuan Jiang

**Affiliations:** 1 Key Laboratory of Automobile Materials, Ministry of Education, Department of Materials Science and Engineering, Jilin University, No. 5988 Renmin Street, Changchun 130025, China; tiantong1990@gmail.com (W.-S.T.); zcjqiuzhi@163.com (C.-J.Z.); qiufeng@jlu.edu.cn (F.Q.); 2 State Key Laboratory of Automotive Simulation and Control, Jilin University, No. 5988 Renmin Street, Changchun 130025, China

**Keywords:** metal matrix composites, nano-sized TiC_p_, elevated temperature dry sliding wear

## Abstract

Nano-sized ceramic particle reinforced aluminum composites exhibit excellent room-temperature mechanical properties. However, there is limited research on the dry sliding wear behavior of those composites at elevated temperatures, which should be one of the major concerns on elevated temperature applications. Here the Al-Cu composites reinforced with nano-sized TiC_p_ were fabricated. The dry sliding wear behaviors of the nano-sized TiC_p_/Al-Cu composites at various temperatures (140–220 °C) and loads (10–40 N) with different TiC_p_ contents were studied, and the results showed that the nanocomposites exhibited superior wear resistance. For instance, the relative wear resistance of the 0.5 wt.% nano-sized TiC_p_/Al-Cu composite was 83.5% higher than that of the Al-Cu matrix alloy at 180 °C under 20 N, and was also 16.5% higher than that of the 5 wt.% micro-sized TiC_p_/Al-Cu composite, attributed to the pronounced Orowan strengthening effect of nanoparticles. The wear rates of the nanocomposites were always lower than those of the Al-Cu matrix alloy under the same test condition, which increased with the increase in temperature and load and with the decrease in TiC_p_ content.

## 1. Introduction

Aluminum alloys have relatively high room-temperature strength. However, the reduced mechanical properties at elevated temperatures limit their applications. For example, some high-speed reciprocating or rotating components (e.g., pistons, drive shafts and brake rotors) often operate at temperature higher than 140 °C, where dry sliding would happen if oil starvation or adverse operating conditions arise [1]. One way to improve the elevate temperature mechanical properties of Al alloys is to introduce various particles into the Al matrix alloys. The particle reinforcements (such as TiC [1,2,3], ZrSiO_4_ [4], TiB_2_ [5,6], ZrB_2_ [7], B_4_C [8] and SiC [9,10]) provide isotropic strengthening effect and cost-effectiveness. The wear behavior of the particle reinforced Al matrix composites at elevated temperatures is one of the major concerns for elevated temperature applications. The high-temperature wear behaviors of micro-sized particle reinforced metal matrix composites (microcomposites) have been reported in the literature [5,11,12]. Natarajan et al. [11] showed that the relative wear resistance of the 5.0 micro-sized TiB_2_/6063 composite fabricated by the flux assisted synthesis approach was 9.0% higher than that of the matrix alloy in dry sliding wear tests at 200 °C under 9.8 N applied load. However, there is a lack of published work on the dry sliding wear behavior of nanocomposites at elevated temperatures and comparison the effect of nano-sized particles with micro-sized particles on the elevated temperature wear behavior of Al matrix composites. The effect of the particle size on the room-temperature dry sliding wear behavior of the particle reinforced metal matrix composites against the steel counterface has been reported [3,9,13]. Mahdavi et al. [13] found that the increased SiC particle size from 19 μm to 146 μm resulted in higher wear resistance of the SiC_p_/Al6061 composites at room temperature. However, if the particle size is reduced to nanometer scale, the nano-sized particle reinforced metal matrix composites (nanocomposites) exhibit better room-temperature tribological properties than the microcomposites. Nemati et al. [3] found that the relative wear resistance of the 5.0 wt.% nano-sized TiC_p_/Al-4.5Cu composite produced by the powder metallurgy technique was 88.7% and 41.1% higher than that of the matrix alloy and the 5.0 wt.% micro-sized TiC_p_/Al-4.5Cu composite in the room-temperature dry sliding wear tests under 20 N applied load, respectively. Moazami-Goudarzi et al. [9] reported that the 5.0 wt.% nano-sized SiC_p_/Al5252 composite which were produced using powder metallurgy showed 211.8% and 120.4% higher relative wear resistance than the matrix alloy and the 10 wt.% micro-sized SiC_p_/Al5252 composite in the room-temperature dry sliding wear tests under 45 N load. Although studies exploring the room temperature wear behaviors of Al matrix nanocomposites are numerous, research on their elevated temperature wear behaviors is very limited. Therefore, in this paper, an effort has been made to bridge this gap.

Among the various ceramic reinforcement particles, TiC is attractive due to its outstanding properties, such as high hardness (2895–3200 HV [14]), low density (4.93 g/cm^3^ [2]), high melting temperature (3250 °C [14]), high modulus (269 GPa [2]), excellent wear resistance and good wettability with molten aluminum [15]. Casting is a popular method to fabricate metal matrix composites, owing to cost effectiveness and near net shaping [4]. In our previous work [16,17], the addition of the 0.5 wt.% nano-sized TiC_p_ by casing method could significantly improve the strength and ductility of an Al-Cu alloy at room temperature. It seems promising that the addition of nano-sized TiC_p_ would improve its elevated temperature wear properties. However, to our knowledge, the elevated temperature wear resistance of nano-sized TiC_p_ reinforced Al-Cu matrix composites has not been reported.

Therefore, in the present study the elevated temperature dry sliding wear properties of the nano-sized TiC_p_/Al-Cu composites at different temperatures under various applied loads were investigated and compared with the micro-sized TiC_p_/Al-Cu composites at 180 °C under 20 N load. The wear mechanisms and the strengthening mechanisms were also investigated. This work aims to provide a new approach to increase the elevated temperature wear resistance of Al matrix composites.

## 2. Experimental Procedures

The Al-Cu alloy with a chemical composition in mass% of Al-5Cu-0.45Mn-0.3Ti-0.2Cd-0.2V-0.15Zr-0.04B was chosen as the matrix alloy of the composites. 70Al-Ti-carbon nanotubes/30Al-Ti-carbon black (in wt.%) at a ratio corresponding to that of stoichiometric TiC was used as the self-propagation high-temperature synthesis (SHS) reaction system to produce nano-sized/micro-sized TiC_p_-Al master alloy in a self-made vacuum thermal explosion furnace at 900 °C, respectively [18,19]. The composites containing 0.1–1.0 wt.% nano-sized TiC_p_ and 1.0–5.0 wt.% micro-sized TiC_p_ were casted by adding different master alloys into molten Al-Cu alloys followed by mechanical stirring. For more details of the fabrication process, readers can refer to [16].

The master alloys were dissolved in an 18 vol.% HCl water solution and were dried in air to observe the morphologies and sizes of the nano-sized and micro-sized TiC_p_. The microstructures were investigated by optical microscopy (Axio Imager A2m, Zeiss, Oberkochen, Germany), field emission scanning electron microscope (FESEM, JSM 6700F, Tokyo, Japan), transmission electron microscope (TEM, JEM 2100F, Tokyo, Japan) and scanning electron microscopy (SEM, Evo18, Zeiss, Oberkochen, Germany). The phase compositions in both the nano-sized and micro-sized TiC_p_-Al master alloys were identified by X-ray diffraction (XRD, Rigaku D/Max 2500PC, Tokyo, Japan) with CuKα radiation using a scanning speed of 4 °/min.

After T6 heat treatment (solution treatment at 538 °C for 12 h and aging at 165 °C for 10 h), the composites were machined to cylindrical pins with a diameter of 6 mm and a height of 12 mm. Vickers hardness measurements were performed at room temperature with a load of 1 kg and a dwell time of 15 s. Dry sliding wear tests were carried out using a pin-on-disc wear apparatus (MG2000, Zhangjiakou, China). A steel disc (H13 steel) with the hardness of 50 HRC and a diameter of 70 mm was used as the counter disc. Both the samples and disc were polished mechanically using the emery paper with an average grit size of 5 μm before each test, and were placed inside a furnace chamber. The sliding velocity and test time were kept constant in all the wear tests at 200 r/min and 10 min, respectively. The wear tests were carried out at various temperatures (140, 180 and 220 °C) and loads (10, 20, 30 and 40 N). The test temperature was measured by a thermocouple located inside the furnace chamber. Each specimen was heated to the setting temperature in the furnace chamber and held for 15 min at the isothermal condition before the wear test. The weight loss was measured using a microbalance with an accuracy of 0.0001 g and then converted into volume loss per unit sliding distance to calculate the wear rate. Relative wear resistance, i.e. the ratio of the wear rate of the Al-Cu matrix alloy to that of the composite, was used to evaluate the wear resistance of the materials and the relative wear resistance of the Al-Cu matrix alloy was taken as 1.000. The worn surfaces and wear debris were examined by SEM and the surface roughness of the worn surfaces were examined by stylus profiler (XP-100, Ambios, Santa Cruz, CA, USA).

## 3. Results and Discussion 

### 3.1. Microstructure

**Figure 1:**
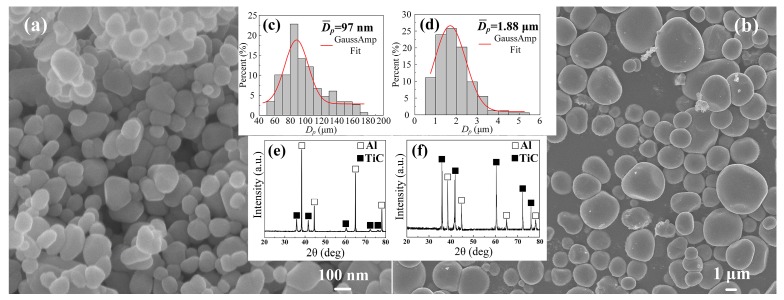
Morphologies and sizes of the TiC_p_ formed in the SHS reaction systems of (**a**) 70Al-Ti-carbon nanotubes and (**b**) 30Al-Ti-carbon black, (**c**,**d**) the corresponding statistical results of the diameters of the TiC_p_ in (**a**,**b**), respectively, (**e**,**f**) the XRD patterns of the nano-sized TiC_p_-Al master alloy and micro-sized TiC_p_-Al master alloy, respectively.

**Figure 2:**
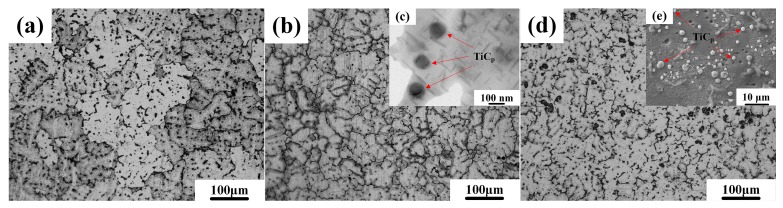
The typical optical microstructures of the as-cast (**a**) Al-Cu matrix alloy, (**b**) 0.5 wt.% nano-sized TiC_p_/Al-Cu composite and (**d**) 5.0 wt.% micro-sized TiC_p_/Al-Cu composite; (**c**) the TEM micrograph of the 0.5 wt.% nano-sized TiC_p_/Al-Cu composite; (**e**) the SEM micrograph of the 5.0 wt.% micro-sized TiC_p_/Al-Cu composite.

Figure 1a,b show the FESEM images of the TiC_p_ extracted from the nano-sized and micro-sized TiC_p_-Al master alloys, respectively. It is evident that the in-situ nano-sized and micro-sized TiC_p_ were near-spherical in morphology with a mean size of about 97 nm and 1.88 μm (Figure 1c,d, respectively. Figure 1c,d show the XRD patterns of the nano-sized TiC_p_-Al master alloy and micro-sized TiC_p_-Al master alloy, respectively. As indicated, the master alloys contained only Al and TiC phases without phases such as Al_3_Ti or Al_4_C_3_. Figure 2 shows the optical as-cast microstructures of the Al-Cu matrix alloy and the composites. The mean size of α-Al grains in the Al-Cu matrix alloy was about 160 μm (Figure 2a). The addition of TiC_p_ reduced the mean size of α-Al grains, which achieved about 65 μm in the 0.5 wt.% nano-sized TiC_p_/Al-Cu composites (Figure 2b) and 75 μm in the 5 wt.% micro-sized TiC_p_/Al-Cu composites (Figure 2d). The TEM micrograph of the nano-sized TiC_p_/Al-Cu composite shows that the nano-sized TiC_p_ were distributed in the grain interior (Figure 2c). The SEM observation of the 5 wt.% micro-sized TiC_p_/Al-Cu composite (Figure 2e) shows many spherical micro-sized particles embedded inside the matrix alloy. The Energy-dispersive X-ray spectroscopy (EDS) analysis verified that those particles were micro-sized TiC_p_. The TiC_p_ were found to be distributed relatively uniformly with limited agglomerations and situated inside the α-Al grains instead of the grain boundaries, due to the good wettability between the in situ TiC_p_ and the aluminum matrix [16]. 

### 3.2. Dry Sliding Wear Behavior of the Nano-sized TiC_p_/Al-Cu Composite 

#### 3.2.1. Effect of Temperature

**Figure 3:**
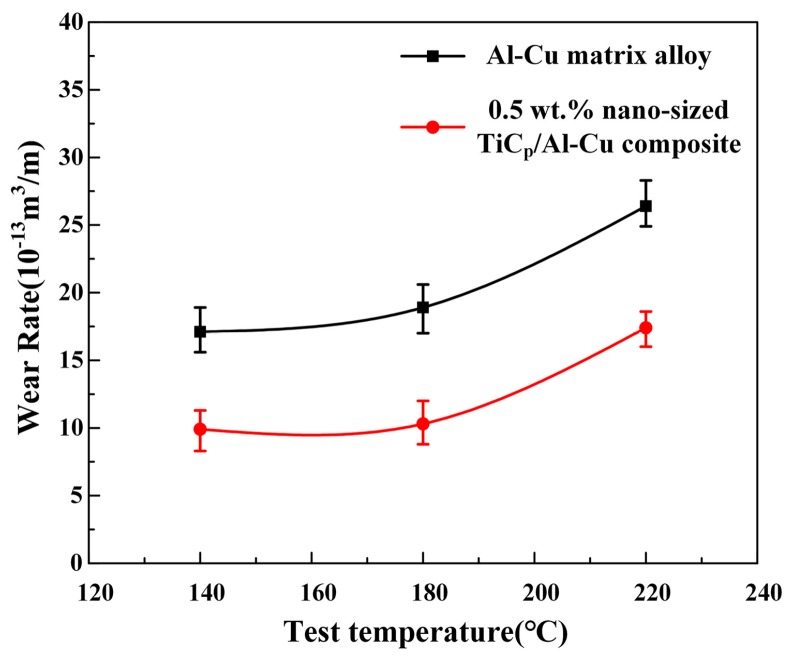
The wear rates of the Al-Cu matrix alloy and 0.5 wt.% nano-sized TiC_p_/Al-Cu composite at different temperatures.

**Figure 4:**
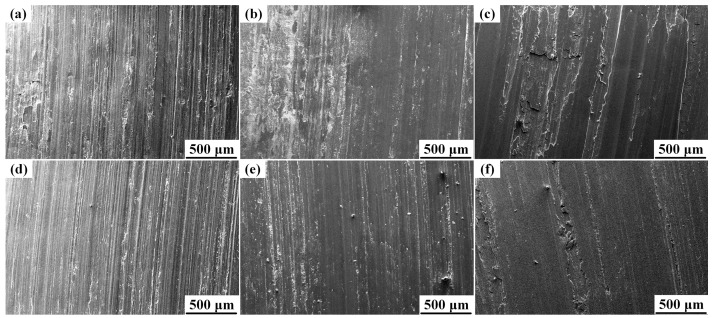
The worn surfaces of the Al-Cu matrix alloy and 0.5 wt.% nano-sized TiC_p_/Al-Cu composite at different temperatures under 20 N load: the Al-Cu matrix alloy at (**a**) 140 °C, (**b**) 180 °C and (**c**) 220 °C; the nanocomposite at (**d**) 140 °C, (**e**) 180 °C and (**f**) 220 °C.

Figure 3 shows the wear rates of the Al-Cu matrix alloy and 0.5 wt.% nano-sized TiC_p_/Al-Cu composite in the temperature range from 140 °C to 220 °C under a constant load of 20 N. It is obvious that the wear resistance of the nanocomposite was superior to that of Al-Cu matrix alloy at all temperatures. The relative wear resistance of the nanocomposite was 72.7%, 83.5% and 51.7% higher than that of the Al-Cu matrix alloy at 140 °C, 180 °C and 220 °C, respectively. Besides, the wear rates of both the matrix alloy and nanocomposite tended to increase with increasing test temperature, and the wear rate increment was small from 140 °C to 180 °C, but a steep increase was observed from 180 °C to 220 °C. Figure 4 shows the worn surfaces of both the Al-Cu matrix alloy and nanocomposite at different temperatures. When the sliding temperature was 140 °C, the worn surface of the matrix alloy was comprised of distinct parallel grooves and minor delamination, while that of the nanocomposite showed only shallower grooves. It indicates that the wear mode at 140 °C for the Al-Cu matrix alloy was a combination of ploughing and delamination while ploughing was dominant for the nanocomposite (Figure 4a,d). With the increase of temperature, the grooves of both matrix alloy and nanocomposite became shallower. The grooves were formed on the soft materials (Al-Cu alloy and composites) by the ploughing of hard asperities. It is reported that the ductility of the Al-Cu alloy increased with increasing temperatures [20]. During the wear test at elevated temperature, the soft metal which was ploughed tended to flow back, resulting in the shallower grooves at higher temperatures [5]. This result is consistent with other works [5,21]. At 220 °C, the worn surfaces of both the matrix alloy and nanocomposite exhibited obvious delamination, leading to higher wear rates, while the delaminated area of the matrix alloy was much larger than that of the nanocomposite, as seen in Figure 4c,f. Therefore, in this work, the onset temperature of obvious delamination of the nanocomposite (220 °C) was higher than that of the matrix alloy (not higher than 140 °C), indicating the better wear resistance of the nanocomposite. Sliding temperature had two contrary effects on the wear behavior of Al alloys. On the one hand, oxidation at elevated temperatures led to the formation of a thick protective oxide layer on the worn surface, which could reduce the wear rate [1]. On the other hand, the softening of the matrix alloy with the increase of sliding temperature could increase the wear rate [5]. In the range of 140–180 °C, the protective layer could be strong enough to counteract the thermal softening effect of the matrix alloy, which led to little increase of wear rate. At 220 °C, however, the worn surface softened so severely that the oxide layer was detached and the bulk metal was transferred from the pin to the counterface (see Figure 4c,f), giving rise to the significant increase in wear rate. Besides, the presence of nano-sized TiC_p_ in the nanocomposite was expected to improve the softening resistance of the Al-Cu alloy, resulting in the better elevated temperature wear resistance [21].

**Figure 5:**
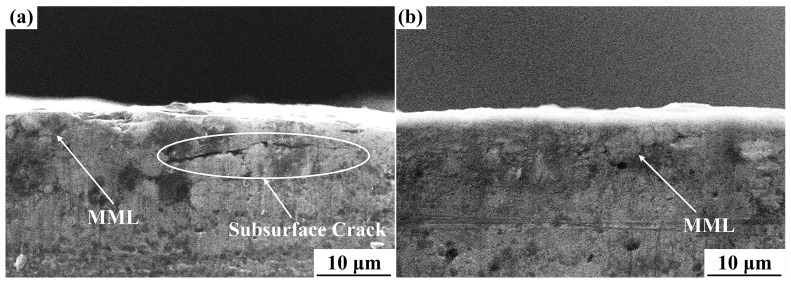
Cross sections normal to the worn surface of (**a**) Al-Cu matrix alloy and (**b**) 0.5 wt.% nano-sized TiC_p_/Al-Cu composite at 180 °C under 20 N load.

EDS analysis of the worn surfaces confirmed the presence of Fe and O in both the Al-Cu matrix alloy and the nanocomposite. The existence of Fe on the worn surface indicated the transfer of Fe from the steel disc to the worn surface, which could be attributed to the abrasive wear mechanism [22]. The abrasive wear was reduced with the increase of temperature, leading to the decreased Fe content. The Fe contents on the worn surfaces of the Al-Cu matrix alloy and nanocomposite tested at 140, 180 and 220 °C under 20 N load were 5.41, 3.31, 1.81 at.% and 5.80, 4.23, 2.58 at.%, respectively. The higher hardness of the nanocomposite (173.5 Hv for the nanocomposite and 158.2 Hv for the Al-Cu matrix alloy at room temperature) resulted in the increased possibility of abrasive action of steel asperities by ploughing into the steel disc and thus increased the Fe transfer. The existence of O indicated the oxidation reaction. During the sliding process, a protective layer called mechanically mixed layer (MML) was formed on the worn surface, which was much harder than the Al-Cu matrix and thus reduced the wear rate [9,23,24]. Figure 5 shows that MML existed in the worn surfaces of both the Al-Cu matrix alloy and nanocomposite tested at 180 °C. A subsurface crack was observed in the Al-Cu matrix alloy (Figure 5a), which would grow and lead to the removal of a layer of metal by delamination wear. However, no evidence of the subsurface crack was observed in the nanocomposite (see Figure 5b).

#### 3.2.2. Effect of Applied Load

**Figure 6:**
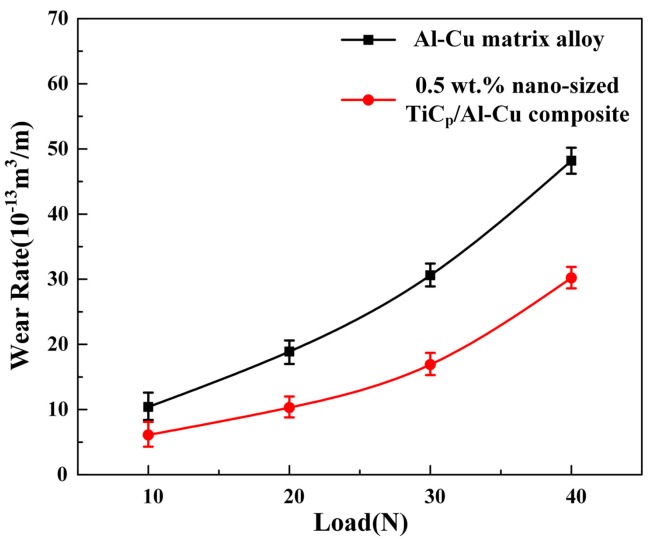
The wear rates of the Al-Cu matrix alloy and 0.5 wt.% nano-sized TiC_p_/Al-Cu composite under different loads at 180 °C.

**Figure 7:**
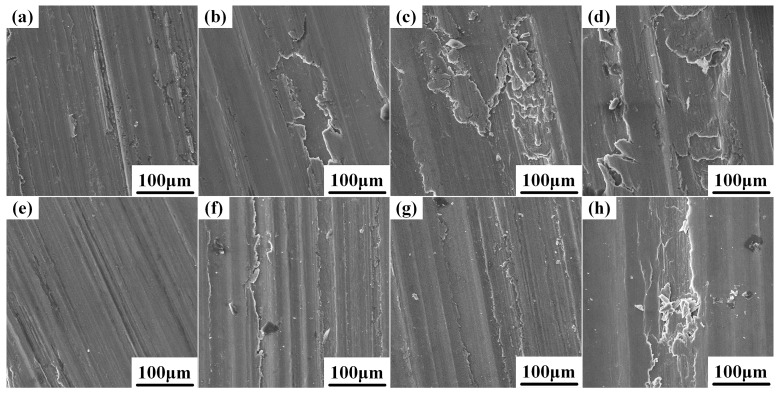
The worn surfaces of the Al-Cu matrix alloy and 0.5 wt.% nano-sized TiC_p_/Al-Cu composite under different loads at 180 °C: the Al-Cu matrix alloy (**a**–**d**): 10 N, 20 N, 30 N and 40 N; the nano-sized TiC_p_/Al-Cu composite (**e**–**h**): 10 N, 20 N, 30 N and 40 N.

The wear rate at 180 °C under the load range from 10 N to 40 N is shown in Figure 6. The wear rates of the Al-Cu matrix alloy and 0.5 wt.% nano-sized TiC_p_/Al-Cu composite increased with the increase of applied load. The nanocomposite exhibited superior wear resistance in the load range of 10 N–40 N, of which the relative wear resistance was improved by 59.6%–83.5%, compared with the Al-Cu matrix alloy. Figure 7 shows the worn surfaces of both the Al-Cu matrix alloy and the nanocomposite at different loads. At 10 N load, the worn surface of the Al-Cu matrix alloy exhibited relatively smooth surface with shallow parallel grooves, as shown in Figure 7a. As the load increased to 20 N and above, the surface morphology of the worn surface changed from distinct grooves to heavy delamination, leading to a rapid increase in the wear rate (see Figure 6 and Figure 7b,d). However, the worn surface of the nanocomposite showed grooves with relatively small width and depth and little delamination at 10–30 N load, compared with the matrix alloy, as seen in Figure 7e–g. At 40 N load, delamination was observed in the worn surface of the nanocomposite (Figure 7h), leading to a rapid increase in the wear rate (Figure 6), but the delaminated area of the nanocomposite was smaller than that of the matrix alloy. Increasing the applied load could result in higher extent of plastic deformation of the soft matrix and the matrix would be ploughed deeper to create larger grooves in the worn surface. Furthermore, large load would lead to the onset of delamination and larger material was removed from the surface [25], but the onset load of delamination of the nanocomposite (40 N) was higher than that of the Al-Cu matrix alloy (20 N), indicating the better high temperature wear resistance of the nanocomposite. Therefore, the wear rates of both the matrix alloy and nanocomposite were higher at larger load.

#### 3.2.3. Effect of TiC_p_ Contents

**Figure 8:**
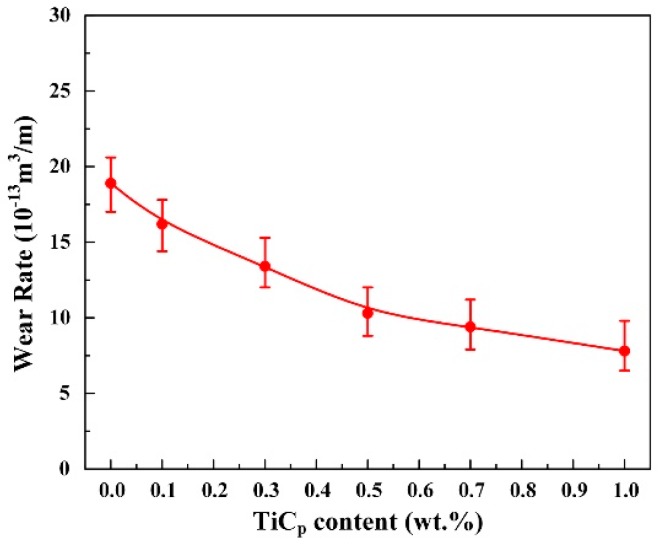
The wear rates of the Al-Cu matrix alloy and nano-sized TiC_p_/Al-Cu composites reinforced by different contents of nano-sized TiC_p_ under 20 N load at 180 °C.

**Figure 9:**
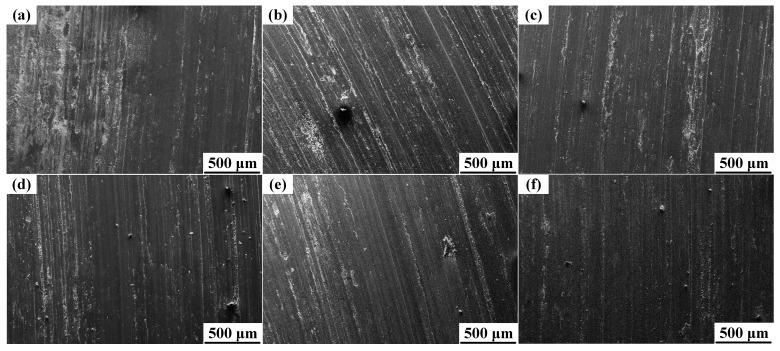
The worn surfaces of (**a**) the Al-Cu matrix alloy and nano-sized TiC_p_/Al-Cu composite reinforced by different contents of nano-sized TiC_p_ under 20 N load at 180 °C: (**b**) 0.1 wt.%, (**c**) 0.3 wt.%, (**d**) 0.5 wt.%, (**e**) 0.7 wt.% and (**f**) 1.0 wt.%.

Figure 8 exhibits the variation of wear rates with the contents of nano-sized TiC_p_ under 20 N load at 180 °C. The wear rate (10^−13^ m^3^/m) of the Al-Cu matrix alloy was 18.9. By adding the 0.1 wt.% nano-sized TiC_p_, the wear rate decreased to 16.2, and the relative wear resistance was improved by 17.0%. With the increase in the content of nano-sized TiC_p_, the wear rate tended to decrease and the relative wear resistance tend to increase. The wear rate (10^−13^ m^3^/m) and the relative wear resistance of the 1.0 wt.% nano-sized TiC_p_/Al-Cu composite were 7.8 and 2.42, respectively, which were decreased by 58.7% and increased by 242.3%, compared with the Al-Cu matrix alloy. Figure 9 shows the worn surface under 20 N load at 180 °C of the Al-Cu matrix alloy and the nano-sized TiC_p_/Al-Cu composite reinforced by different contents of nano-sized TiC_p_. As seen from Figure 9a, the worn surface of Al-Cu matrix alloy was comprised of parallel grooves and delaminated areas. However, with the addition of nano-sized TiC_p_, the patches of material removal became smaller and shallower, as shown in Figure 9b–f. With the increase of the content of nano-sized TiC_p_, the width and depth of the parallel grooves tended to decrease and the worn surfaces were becoming smooth. Besides, in the nano-sized TiC_p_/Al-Cu composites, little or none delamination was observed in the worn surfaces, indicating the better wear resistance of the nanocomposites.

### 3.3. Comparing with the Micro-Sized TiC_p_/Al-Cu Composite 

Table 1 lists the wear rates of the Al-Cu matrix alloy, 1.0–5.0 wt.% micro-sized TiC_p_/Al-Cu composite and 0.5 wt.% nano-sized TiC_p_/Al-Cu composite at 180 °C under a constant load of 20 N. As shown in Table 1, the wear resistance of the composites was much higher in comparison to the unreinforced Al-Cu matrix alloy. Besides, the wear resistance of the microcomposites increased with the increasing micro-sized TiC_p_ content. The relative wear resistance of the 5.0 wt.% micro-sized TiC_p_/Al-Cu composite was improved by 57.5% compared with the Al-Cu alloy. The nanocomposite exhibited the highest relative wear resistance, which improved by 83.5% and 16.5%, compared with the Al-Cu alloy and 5.0 wt.% micro-sized TiC_p_/Al-Cu composite, respectively, although the mass fraction of the nano-sized TiC_p_ (0.5%) was much lower than that of the micro-sized TiC_p_ (5%). The improvement in the wear resistance of the composites compared with the matrix alloy was more significant than that of the 5.0 micro-sized TiB_2_/6063 composite (9% at 200 °C under 9.8 N applied load) fabricated by the flux assisted synthesis approach reported by Natarajan et al. [11]. It indicates that the nano-sized reinforcement TiC_p_ was very effective to improve the elevated temperature wear resistance of the Al matrix composites. The Vickers hardness of the Al-Cu matrix alloy (158.2 Hv), 5.0 wt.% micro-sized TiC_p_/Al-Cu composite (166.6 Hv) and 0.5 wt.% nano-sized TiC_p_/Al-Cu composite (173.5 Hv) were measured. It is obvious that as the hardness increased, the wear rates decreased. This result is consistent with the well-known Archard’s equation in which the wear volume is inversely proportional to the hardness of the worn material [26,27]. In the alloys with lower hardness, larger plastic deformation of surface and subsurface during sliding caused more delamination and production of a higher volume of wear debris, leading to the increased surface roughness and higher wear rate [27].

**Figure 10:**
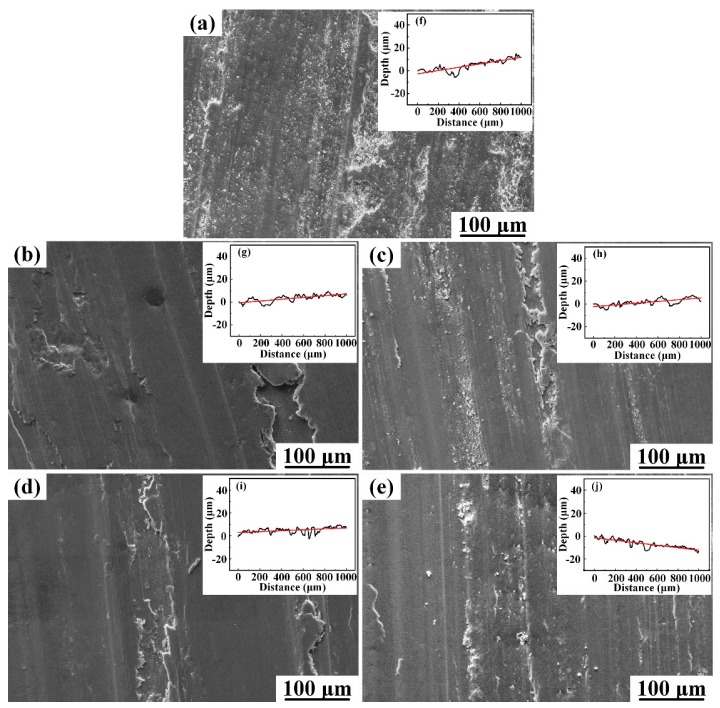
The worn surfaces of (**a**) the Al-Cu matrix alloy and Al-Cu matrix composites reinforced with different contents of micro-sized TiC_p_ ((**b**) 1.0 wt.%, (**c**) 3.0 wt.%, (**d**) 5.0 wt.%) and (**e**) 0.5 wt.% nano-sized TiC_p_ at 180 °C under a constant load of 20 N; (**f**–**j**) corresponding roughness curves of the worn surface.

**Figure 11:**
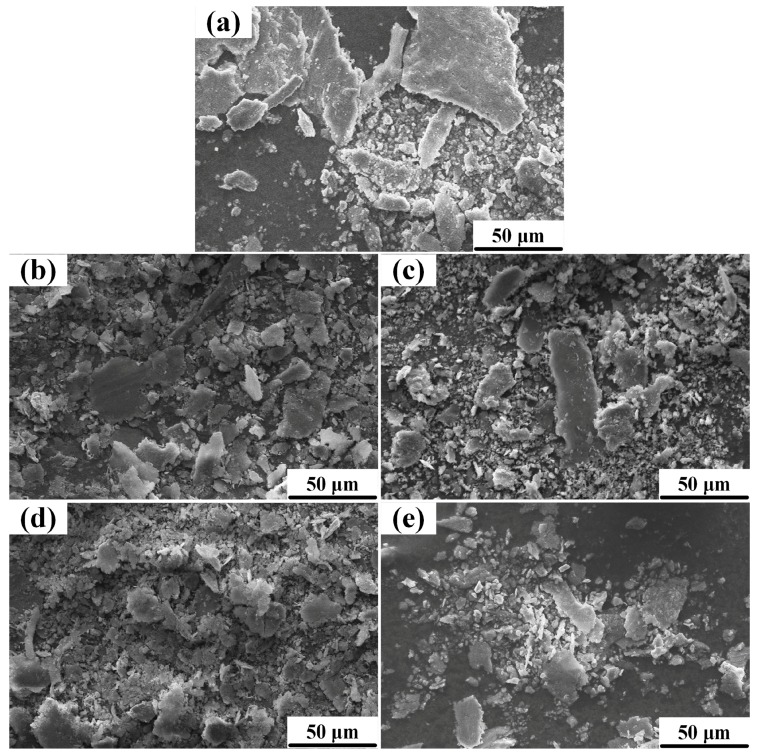
The wear debris morphologies of (**a**) the Al-Cu matrix alloy and Al-Cu matrix composites reinforced with different contents of micro-sized TiC_p_: ((**b**) 1.0 wt.%, (**c**) 3.0 wt.%, (**d**) 5.0 wt.%) and (**e**) 0.5 wt.% nano-sized TiC_p_ at 180 °C under a constant load of 20 N.

Figure 10a–e show the SEM micrographs of worn surfaces of the Al-Cu matrix alloy, microcomposites and nanocomposite. Figure 10f–j show the surface roughness profiles of the samples after wear tests, and Table 1 presents the arithmetic average height (*R_a_*) of the profiles, which is defined as the average absolute deviation of the roughness irregularities from the mean line over a sampling length [28]. As seen from Figure 10a, the worn surface of the Al-Cu matrix alloy was comprised of parallel grooves and large delaminated areas. The worn surface profile of the Al-Cu matrix alloy undulated greatly, and the grooves were relatively large in width and depth (see Figure 10f). Figure 11a shows the wear debris generated from the Al-Cu matrix alloy. The wear debris had an irregular shape and were large in size (more than 50 μm), which was ploughed from the large delaminated areas. However, with the increase of the micro-sized TiC_p_ content, the delaminated area became smaller and shallower, and the roughness profile became smoother and the R_a_ value decreased, as shown in Figure 10b–d,g–i. This led to the creation of smaller wear debris, as shown in Figure 11b–d. In the nanocomposite, no delaminated area was observed. The worn surface was relatively smooth and the depth of parallel narrow grooves was shallow, the roughness profile showed a smoothest appearance with the lowest *R_a_* value of 1.501 μm, decreased by 21.7% and 8.5%, compared with the matrix alloy and 5 wt.% micro-sized TiC_p_/Al-Cu composite, respectively, indicating that the nanocomposite exhibited the best wear resistance. The absence of delamination wear in nanocomposite could be due to the stronger interfacial bond between nano-sized particles and Al matrix than that between micro-sized particles and Al matrix. It is reported that in the composites reinforced with micro-sized particles larger than 1 μm, cracks are more likely to be nucleated at the particle/matrix interface during sliding wear than in the nanocomposites [9]. As a result, the micro-sized TiC_p_/Al-Cu composites exhibited more wear volume loss due to delamination wear while the nanocomposite resisted delamination and showed better wear resistance. Figure 11e shows that the wear debris created from the nanocomposite was much smaller than that created from the Al-Cu matrix alloy and microcomposites, leading to the less volume loss.

TiC_p_ have two effects on the elevated temperature wear behavior, that is, strengthening effect and protective effect. It is reported that the wear rate of a material was inversely proportional to its yield strength [29]. Therefore, the improvement in the strength can also enhance the wear resistance. Firstly, it is reported that Orowan strengthening of ceramic particles is much more significant in nanocomposites than in microcomposites [30]. The increment of yield strength at 180 °C caused by Orowan strengthening mechanism (Δ*σ_Oro_*) can be calculated by the following equation [31]:

(1) $$\Delta \sigma_{\mathrm{Oro}}=0.81\frac{\mathrm{MAGb}}{2\pi \lambda }\ln(\frac{\pi d}{4b})$$

where *M* = 3 is the Taylor factor, *G* is the shear modulus of aluminum (*G* = 24.2 GPa at 180 °C according to [32]), *b* = 0.286 nm is the Burgers vector, *λ* $=0.4d(\sqrt{\pi /\mathrm{fV}}-2)$ is the interparticle spacing, *d* and *f_V_* are the diameter and volume fraction of the TiC_p_, respectively. The value of constant *A* is estimated to be 1.8 [31]. The theoretical value of Δ*σ_Oro_* in the nanocomposite is 21.8 MPa, much higher than that of the microcomposite (2.5–6.4 MPa). Secondly, efficient transfer of load from the Al-Cu matrix to the stiffer TiC_p_ can also contribute to the increase of yield strength. Compared with the Al-Cu matrix alloy, the increase in the yield strength of the composites caused by load transfer effect at 180 °C can be estimated using the following equation [33]:

Δ*σ_Load_* = 0.5*f_V_*σ*_ym_* (2)

where *f_V_* is the volume fraction of the TiC_p_ and *σ_ym_* is the yield strength of the Al-Cu matrix alloy at 180 °C, which equals 221 MPa according to [20]. The calculated Δ*σ_Load_* in the 5 wt.% micro-sized TiC_p_/Al-Cu composite is 3.1 MPa, while in the nanocomposite the result is 0.3 MPa. The calculated results of Δ*σ_Oro_* and Δ*σ_Load_* are listed in Table 1. It is obvious that the contribution of the nano-sized TiC_p_ to yield strength at 180 °C is more significant than that of the micro-sized TiC_p_, attributed to the pronounced Orowan strengthening effect of nanoparticles. Thirdly, it is found that the wear resistance and grain size of pure aluminum follow a relationship identical to Hall-Petch effect [34]. The grain refinement contributes to the improvement of wear resistance of both the nanocomposite and the microcomposite compared with the Al-Cu matrix alloy. However, the grain refinement effect is negligible for the comparison between the nanocomposite and microcomposite owing to the little difference in grain size. Besides, grain refinement of the Al-Cu matrix alloy leads to a larger number and finer θ′ precipitates after aging, resulting in an enhanced precipitation strengthening effect [16]. Similarly, the precipitation strengthening effect is negligible for the comparison between the nanocomposite and microcomposite due to their similar grain sizes and the rapid coarsening of θ′ precipitates at elevated temperatures [20]. Therefore, the yield strength of the nanocomposite was much higher than that of the microcomposite due to the pronounced Orowan strengthening effect of nanoparticles, leading to higher wear resistance at high temperature.

Meanwhile, our previous study found that the nano-sized TiC_p_ bonded well with the Al matrix [16]. During sliding, the Al matrix surrounding the nano-sized particles was worn away and the contact was provided between the nano-sized particles and the steel counter face. Therefore, the presence of the nano-sized hard ceramic particles could protect the Al-Cu matrix from direct contact with the counter face, resulting in the better wear resistance of the nanocomposite [35].

## 4. Conclusions

1. The relative wear resistance of the 0.5 wt.% nano-sized TiC_p_/Al-Cu composite was 72.7%, 83.5% and 51.7% higher than that of the Al-Cu matrix alloy at 140 °C, 180 °C and 220 °C under 20 N load, respectively. The wear rate of the nanocomposite increased with the increase in temperature, but it was still lower than that of the Al-Cu matrix alloy at the same temperature. The worn surfaces indicated that the dominant wear mode for the nanocomposite was ploughing at 140 °C and 180 °C, and a combination of ploughing and delamination at 220 °C, while for the matrix alloy a combination of ploughing and delamination was dominant at all sliding temperatures studied.
2. The nanocomposite exhibited superior wear resistance in the load range of 10 N–40 N at 180 °C, of which the relative wear resistance was improved by 59.6–83.5%, compared with the Al-Cu matrix alloy. The wear rate of the nanocomposite increased with the increase in the load, similarly to the Al-Cu matrix alloy, while it was lower than that of the matrix alloy under the same load. The worn surfaces indicated that the onset load of obvious delamination of the nanocomposite (40 N) was higher than that of the Al-Cu matrix alloy (20 N). Besides, with the increase in the content of nano-sized TiC_p_, the relative wear resistance tended to increase.
3. The 0.5 wt.% nano-sized TiC_p_/Al-Cu composite exhibited superior high-temperature dry sliding wear resistance to the 5 wt.% micro-sized TiC_p_/Al-Cu composite at 180 °C under a constant load of 20 N, of which the relative wear resistance was 16.5% higher, attributed to the pronounced Orowan strengthening effect of nanoparticles in the nanocomposite.

## Figures and Tables

**Table 1:** The high-temperature wear properties and the theoretical values of Δ*σ_Oro_* and Δ*σ_Load_* at 180 °C of the Al-Cu alloy and TiC_p_/Al-Cu composites with different sizes and contents of TiC_p_.

Samples (wt.%)	Wear Rate (10^−13^ m^3^/m)	Relative Wear Resistance	Surface Roughness *R_a_* (μm)	Δ*σ_Oro_ +* Δ*σ_Load_* (MPa)
Al-Cu alloy (M)	$18.9\begin{array}{c} +1.7\\ -1.9 \end{array}$	1.000	1.918	0.0
M+1.0 micro-TiC_p_	$14.1\begin{array}{c} +1.7\\ -1.4 \end{array}$	1.340	1.812	2.5 + 0.6
M+3.0 micro-TiC_p_	$13.3\begin{array}{c} +1.8\\ -1.6 \end{array}$	1.421	1.706	4.7 + 1.8
M+5.0 micro-TiC_p_	$12.0\begin{array}{c} +1.9\\ -1.5 \end{array}$	1.575	1.640	6.4 + 3.1
M+0.5 nano-TiC_p_	$10.3\begin{array}{c} +1.7\\ -1.5 \end{array}$	1.835	1.501	21.8 + 0.3
